# Differences in lower limb co-contraction calculations vary clinical interpretation of aquatic treadmill walking in typically developing and children with cerebral palsy

**DOI:** 10.3389/fneur.2025.1506326

**Published:** 2025-02-27

**Authors:** Joseph W. Harrington, Brian A. Knarr, Vivek Dutt, David C. Kingston

**Affiliations:** ^1^Department of Biomechanics, University of Nebraska, Omaha, NE, United States; ^2^Department of Orthopaedic Surgery & Rehabilitation, University of Nebraska Medical Center, Omaha, NE, United States

**Keywords:** electromyography, CCI, muscle activity, pediatrics, gait

## Abstract

**Objective:**

The purposes of this study were to (1) investigate muscle co-contraction during aquatic (Wet) and conventional (Dry) treadmill walking at various speeds in typically developing (TD) and children with cerebral palsy (CP) and (2) explore how the clinical interpretation of co-contraction, using co-contraction indices (CCI), may vary depending on the method employed.

**Methods:**

Fifteen TD children (30 limbs, 7 M | 8F, 11.3 ± 4.1 yrs., 1.46 ± 0.18 m, 44.2 ± 16.8 kg) and 10 children with CP (20 limbs, 6 M | 4F, 13.1 ± 3.5 yrs., 1.54 ± 0.18 m, 53.2 ± 26.2 kg, 7 GMFCS I and 3 II) participated in this study. Muscle activity of the tibialis anterior (TA), rectus femoris (RF), medial gastrocnemius (MG), and semitendinosus (ST) was recorded during three 3-min walking trials on a Dry treadmill followed by a Wet treadmill. Muscle co-contraction was calculated using three common CCI calculation methods for the RF/ST and TA/MG muscle pairings. Separate linear mixed-effects models examined the influence of population (TD vs. CP), walking speed (Slow, Normal, Fast), and treadmill environment (Dry vs. Wet) on CCI for each equation and muscle pairing.

**Results:**

CCI_Unnithan_ and CCI_Rudolph_ demonstrated that aquatic treadmill walking reduced muscle co-contraction in TD (*p* < 0.001) and CP (*p* < 0.012) populations for the RF/ST muscle pairing. Additionally, CCI_Unnithan_ and CCI_Rudolph_ showed significant differences between speeds in both environments (*p* < 0.001) except for the Slow-Normal comparison in the aquatic treadmill (*p* > 0.423). All methods had a significant CCI reduction in the TA/MG muscle pairing for both populations. For the RF/ST muscle pairing, CCI_F&W_ showed that only TD children had lower muscle co-contraction in the aquatic treadmill (*p* = 0.023). CCI_F&W_ also showed no speed effect for the muscle pairings.

**Conclusion:**

This study shows the potential of aquatic treadmill walking to reduce muscle co-contraction; however, caution is recommended as clinical implications can vary due to the computation method. Future studies should aim to report values from multiple methods to account for the variability within methods and validation of results.

## 1 Introduction

Cerebral palsy (CP) is one of the most prevalent neuromuscular disorders (1) and the most common cause of motor disability in juveniles, with an incidence rate of 3.3 per 1,000 live births (2). Children with CP are often classified by functional capability using scales such as the Gross Motor Function Classification System (GMFCS) (3). Children are divided into five functional levels, where lower levels (e.g., GMFCS I-II) correspond to children with impaired balance and coordination but can typically walk with or without assistive devices. In comparison, higher levels (e.g., GMFCS III-V) correspond to children with more severely limited functional mobility, if any. Regardless of functional level, walking efficiency is impaired in children with CP due to physical deformities, muscle weakness, spasticity, and diminished selective motor control (4, 5), leading to altered joint kinematics. Due to muscle spasticity, children with CP often have increased muscle co-contraction and walking impairments that cause increased energy expenditure and reduced walking speed (6). Given these challenges, there is a need for innovative rehabilitation approaches that can not only enhance motor skills and endurance but also have the potential to specifically target improvements in joint range of motion, walking efficiency, and muscle coordination in this clinical population.

Aquatic treadmill walking offers a unique environment to facilitate repetitive gait cycles while capitalizing on the benefits of altered body weight support via buoyancy (7), increased hydrodynamic resistance on the limbs (8), and postural stability. While the hydrodynamic drag of a single human lower leg segment in water was related to limb speed and surface area (8), this relationship remains understudied in children with CP, particularly regarding its impact on gait mechanics. Despite this gap, studies examining the efficacy of aquatic therapy in children with CP have shown promising results in improving gait parameters such as stride length and cadence (9, 10). Furthermore, Phothirook and colleagues investigated muscle co-contraction during overground and aquatic walking in children with CP and typically developing adolescents (11). They found that antagonist hamstring activity was reduced, agonist plantar flexor activity was reduced, and antagonist dorsiflexor activity increased (11). Our group also observed these muscle activation trends in typically developing children during fast aquatic treadmill walking speeds (12). However, inconsistent results (via method of calculating co-contraction) in the literature limit our understanding of muscle co-contraction in children with CP during gait (13) and the potential effects of aquatic treadmill walking in therapeutics remain largely unknown (11, 12).

The co-contraction index (CCI) is a commonly used method that allows clinical researchers to quantify the amount of simultaneous muscle activation during human movements and estimate joint stiffness (14). Generally, muscle co-contraction is defined using the magnitude, duration, or ratio of concurrent activation of functionally opposing muscles about a joint; however, each method has advantages and disadvantages (15). Measuring the magnitude of activation provides a clear indication of muscle intensity but neglects the timing of dynamic movements (16, 17). Focusing on the duration of simultaneous activation gives insights into coordination for a better understanding of movement sequencing but overlooks the intensity of muscle activation (18, 19). A ratio between the magnitude and timing provides a more comprehensive view but can require complex calculations that obscure the directionality of muscle activity about the joint (20, 21). As different methods may yield different interpretations, implications for therapeutic intervention planning are unclear.

By exploring the dynamics of co-contraction during aquatic treadmill walking, clinicians may gain valuable insights into the effectiveness of aquatic treadmill training in addressing muscle co-contraction and spasticity management, thus potentially advancing rehabilitation strategies for children with CP. Therefore, this study aimed to investigate muscle co-contraction during aquatic and conventional treadmill walking at various speeds in typically developing and children with CP. This study calculated co-contraction using three common methods (16, 20, 21) to facilitate comparisons among existing literature. Furthermore, by utilizing multiple CCI calculation methods, we also aimed to explore how clinical interpretation of co-contraction may vary depending on method employed. Based on previous work, we hypothesized that (1) across speeds, co-contraction will be reduced during aquatic treadmill walking (11) and (2) regardless of treadmill environment, increasing walking speed will increase co-contraction (22). These hypotheses were primarily motivated by (1) the increased body weight offloaded in water due to buoyancy and (2) the increased physical demand the faster a person walks due to increased rotational velocity of segments during conventional treadmill walking and hydrodynamic drag during aquatic treadmill walking.

## 2 Materials and methods

### 2.1 Participants

Fifteen typically developing (TD) children (30 limbs, 7 M | 8F, 11.3 ± 4.1 yrs., 1.46 ± 0.18 m, 44.2 ± 16.8 kg) (12) and 10 children with CP (20 limbs, 6 M | 4F, 13.1 ± 3.5 yrs., 1.54 ± 0.18 m, 53.2 ± 26.2 kg, 7 GMFCS I and 3 II) participated in this study. Inclusion criteria for children with CP were (1) aged 6–18, (2) fixed knee flexion deformity exceeding 10° (criterion part of a larger study that encompassed this one), and (3) GMFCS level I-III. Exclusion criteria for children with CP were (1) children that received Botulinum Toxin Type A injections within the past 4 months, and (2) children who cannot independently ambulate with or without assistive devices. Inclusion criteria for TD children were (1) aged 6–18 and (2) no self-reported pain or injuries to the lower limb that required hospitalization within the past 12 months. Exclusion criteria for TD children were (1) children with any lower limb musculoskeletal injury that impairs their ability to walk and (2) medical history of surgical correction for a lower limb injury or deformity. Before study enrollment, all participants and guardians reviewed and signed a letter of informed consent approved by the University of Nebraska Medical Center’s Institutional Review Board.

### 2.2 Experimental procedures

#### 2.2.1 Research design

This study used a block-randomized cross-sectional design wherein participants completed conventional (Dry) treadmill walking trials followed by aquatic (Wet) treadmill walking trials. Each participant attempted three 3-min walking trials on both Dry and Wet treadmills, where the presentation of walking speed was randomized within each treadmill environment. Walking speed was set at 75, 100, and 125% of their self-selected speed, classified as Slow, Normal, and Fast, respectively.

#### 2.2.2 Instrumentation

Participants were instrumented with waterproof wireless surface electromyography (EMG) sensors (MiniWave Waterproof, Cometa, Milan, IT; input impedance = 20 M *Ω*, common mode rejection ratio = 120 dB, bandpass filter 10–1,000 Hz) and inertial measurement unit (IMU) sensors (WaveTrack Waterproof IMU, Cometa, Milan, IT; full-scale acc sensitivity = ± 8 g; full-scale gyroscope sensitivity = 1000dps; dimensions: 36x25x10 mm).

Surface EMG data were recorded at 2000 Hz from the tibialis anterior (TA), medial gastrocnemius (MG), rectus femoris (RF), and semitendinosus (ST). These muscles are assumed to act in agonist/antagonist pairs in sagittal plane movement of the hip, knee, and ankle joints. Electrode sites were located and prepared following SENIAM guidelines (23, 24). Pre-gelled bipolar Ag/AgCl electrodes (sEMG electrodes, Coviden, Dublin, IE) with dimensions of 30 mm by 24 mm and an inter-electrode spacing of 2.5 cm were adhered after shaving and cleaning the skin with alcohol. Raw EMG data were normalized using a dynamic normalization approach similar to den Otter and colleagues (25). Generally, faster walking speeds elicit greater EMG magnitudes, so each participant’s maximum EMG magnitude during the Fast Dry treadmill walking trial was used to normalize muscle activity.

Calibration procedures for the IMUs were conducted according to the manufacturer’s guidelines. First, IMUs were aligned in the same orientation on a table to zero sensors. Next, subjects were instrumented with IMUs on the feet, shanks, thighs, and pelvis. After all instrumentation, a static trial was conducted in which the participant stood in a T-pose with the negative Y-axis of the sensors approximately in line with the participant’s sagittal plane. IMU data were sampled at the maximum of 142 Hz when using the manufacturer’s mixed 6 degrees of freedom (6DoF) sensor-fusion algorithm.

#### 2.2.3 Experimental protocol

The protocol used in this study has been previously described (12). Briefly, the participant’s Normal walking speed was determined for the Dry treadmill. Participants were then instrumented with waterproof wireless surface EMG and IMU sensors. Following instrumentation, participants completed three 3-min walking trials on the Dry treadmill (Precor TRM 835 V2, Precor, Woodinville, WA, United States). Participants then moved to the Aquatic Therapy Lab (~15 m walk) to perform Wet treadmill walking trials, where the water level was set to the participant’s xiphoid process, and the participant’s Normal walking speed was determined again. Participants then completed three 3-min walking trials on the Wet treadmill (300 Series, HydroWorx, Middletown, PA, United States). During each walking trial, participants could stop walking at any point, and the investigator would turn off the treadmill and note the elapsed time.

### 2.3 Data processing

Data processing was completed using MATLAB (2023a, The MathWorks, Natick, MA, United States). Raw EMG data had bias removed, were full wave rectified, and filtered using a dual-pass 2nd order Butterworth filter with a cut-off frequency of 6 Hz to produce a linear envelope (6, 26, 27), and dynamically normalized using the greatest magnitude found in the Dry Fast-walking trial (25). A dynamic normalization approach is common in the literature when calculating CCI (20–22, 28–30). Acceleration data from IMU sensors located on the feet were used to determine gait cycles and events during each walking trial. Initial contacts were determined using the greatest vertical acceleration peaks. Gait cycles (strides) were defined as initial contact to initial contact of the same foot. Any strides beyond three standard deviations of the mean stride time were deemed outliers and removed from the analysis (12). Data from both limbs of all participants were included except in circumstances where equipment malfunction occurred.

The primary outcome variables were the mean CCI of all strides for RF/ST and TA/MG muscle pairings. The co-contraction index was calculated in three ways:


(1) $$CCI_{\mathrm{Unnithan}}=\overset{t_{2}}{\underset{t_{1}}{\int }}\min\left(EMG_{1}\left(t\right),EMG_{2}\left(t\right)\right)\text{.}$$

(2) $$CCI_{\mathrm{Rudolph}}=\frac{EMG\_s\left(t\right)}{EMG\_l\left(t\right)}*\left(EMG\_s\left(t\right)+EMG\_l\left(t\right)\right)\text{.}$$

(3) $$CCI_{F\&W}=\frac{2*EMG\_\mathrm{ant}}{EMG\_\mathrm{total}}*100\%\text{.}$$

For Equation 1, EMG_1_ and EMG_2_ represent the activity of muscle one and muscle two, respectively. Additionally, t_1_ represents 0% stride where t_2_ represents 100%. There is no specific order that the muscles must be for this equation. In simple terms, CCI is calculated as the integral of the common area between the two muscle activation waveforms. For Equations 2, 3, EMG_s, EMG_l, and EMG_ant represent the activity of the smaller, larger, and antagonist muscles, respectively. Co-contraction was time normalized to gait cycle from 0 to 100% of stride. This process was conducted using interpolation to allow comparisons across subjects with varying stride durations. For Equation 2, CCI was calculated at every time *t* from 0 to 100% gait cycle (101 data points). For Equation 3, the antagonist muscle was set as RF for the RF/ST muscle pairing and TA for the TA/MG muscle pairing throughout the gait cycle. For CCI Equations 2, 3, CCI was calculated across the gait cycle to produce 101 data points. The average of those 101 data points was taken to produce a single CCI value for each stride within a trial. For CCI Equation 1, a single CCI value (the area under the curve) was obtained for each stride. For each CCI Eq, the average of all strides’ CCI values within a trial was taken to produce a single CCI value for each trial.

### 2.4 Statistical analyses

Statistical tests were performed using R (RStudio 2022, PBC, Boston, MA, United States) with an *a priori* 𝛼 = 0.05 using the functions lmer() (31) and anova() (32). The lmer() function allows for including fixed and random effects in linear mixed-effects models to account for individual differences across participants and conditions. The anova() function was used to assess the significance of the model’s effects by comparing the variance explained. Separate linear mixed-effects models examined the influence of population (TD vs. CP), walking speed (Slow, Normal, Fast), and treadmill environment (Dry vs. Wet) on CCI for each equation and muscle pairing. Individual differences were accounted for with a random effect of participant. Models were built by incorporating the participant, treadmill environment, population, walking speed, and the interactions among the factors to assess the model fit. Effect sizes were quantified to observe any statistical differences between main and interaction effects. Post-hoc pairwise comparisons using Tukey’s method were calculated to determine statistically significant differences.

## 3 Results

**Figure 1:**
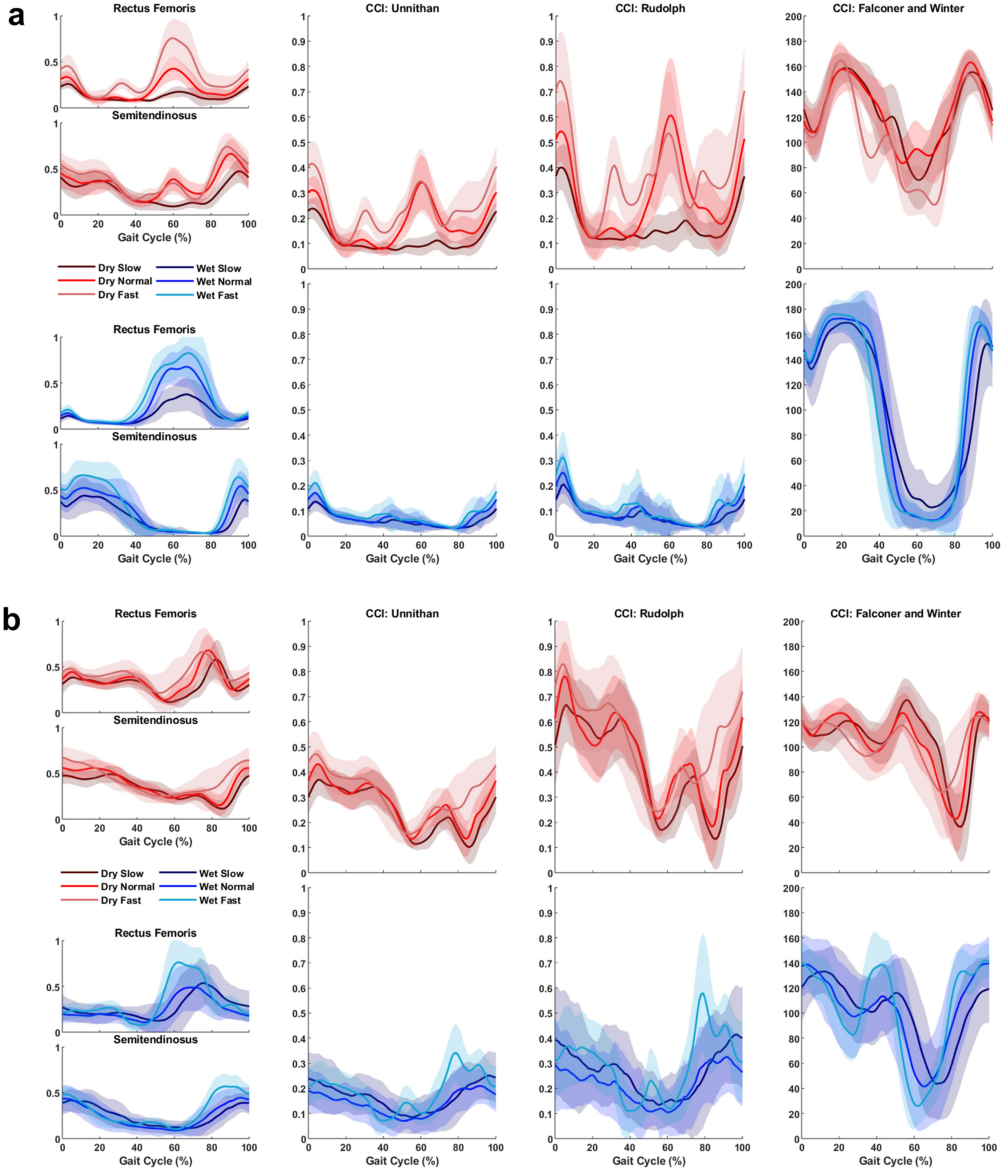
Representative muscle activation and co-contraction index (CCI) changes in one limb for a TD child **(a)** and a child with CP **(b)** across all treadmill conditions and speeds. The leftmost column displays the activation waveforms (after being dynamically normalized) for the muscle pair analyzed, in this scenario Rectus Femoris and Semitendinosus. The middle-left, middle-right, and rightmost columns display CCI changes calculated using Unnithan’s method, Rudolph’s method, and Falconer and Winter’s method, respectively. Each plot shows time series data from 0 to 100% gait cycle, with shades of red lines representing trials on the conventional (Dry) treadmill and shades of blue lines representing trials on the aquatic (Wet) treadmill. Solid lines denote the average across all strides within a trial, while the shaded regions indicate one standard deviation from the mean. Note: The CCI changes using Unnithan’s method correspond to the common activation between the two muscles. The final CCI value would be the integral of those curves.

After outlier removal and data loss, the final sample was 28 limbs for TD children and 15 limbs for children with CP for the TA/MG muscle pairing and 26 limbs for TD children and 14 limbs for children with CP for the RF/ST muscle pairing. Representative RF/ST muscle activation waveforms and CCI changes in one limb for a TD child and a child with CP can be found in Figure 1. The mean and standard deviation of CCI for each muscle pairing and each method of calculation across conditions are reported in Tables 1, 2. Linear mixed-effects models were performed for each muscle pairing and each method of calculating co-contraction, increasing complexity until the model with the lowest Akaike information criterion (AIC), the lowest Bayesian information criterion (BIC), and the greatest log-likelihood (where greater values indicating better fit of the model to the data) with statistical significance noted (Table 3). These metrics were chosen as they are commonly recommended criteria for model comparison and selection throughout the literature (33–36).

**Table 1:** Mean (standard deviation) of stride normalized CCI for the RF/ST muscle pairing in children with CP and TD children during dry and wet treadmill walking at slow, normal, and fast speeds.

		Slow		Normal		Fast	
		Dry	Wet	Dry	Wet	Dry	Wet
CCI_Unnithan_	*CP*	16.66 (6.26)	9.81 (4.66)	19.39 (6.62)	10.43 (4.98)	22.87 (7.52)	14.37 (8.07)
	*TD*	11.84 (4.60)	9.74 (6.61)	14.89 (4.81)	10.81 (7.31)	19.79 (5.42)	13.43 (8.90)
CCI_Rudolph_	*CP*	0.27 (0.11)	0.15 (0.08)	0.31 (0.11)	0.16 (0.08)	0.37 (0.14)	0.22 (0.13)
	*TD*	0.19 (0.08)	0.15 (0.10)	0.24 (0.08)	0.16 (0.11)	0.32 (0.10)	0.20 (0.14)
CCI_F&W_	*CP*	99.98 (14.55)	104.08 (20.32)	96.31 (18.28)	102.63 (19.38)	97.21 (16.97)	98.77 (19.40)
	*TD*	94.42 (20.64)	92.06 (28.98)	95.89 (20.83)	91.56 (30.16)	95.81 (13.00)	90.83 (28.81)

Slow, 75% self-selected walking speed; Normal, 100% self-selected walking speed; Fast, 125% self-selected walking speed; Dry, conventional; Wet, aquatic; CCI_Unnithan_, co-contraction index using the Unnithan method; CCI_Rudolph_, co-contraction index using the Rudolph method; CCI_F&W_, co-contraction index using the Falconer and Winter method; CP, cerebral palsy; TD, typically developing.

**Table 2:** Mean (standard deviation) of stride normalized CCI for the TA/MG muscle pairing in children with CP and TD children during dry and wet treadmill walking at slow, normal, and fast speeds.

		Slow		Normal		Fast	
		Dry	Wet	Dry	Wet	Dry	Wet
CCI_Unnithan_	*CP*	15.70 (5.86)	11.32 (5.56)	17.83 (6.31)	12.82 (6.29)	20.78 (6.73)	16.44 (8.04)
	*TD*	6.22 (1.78)	4.97 (1.76)	7.54 (2.94)	5.75 (1.88)	9.90 (4.04)	7.76 (3.72)
CCI_Rudolph_	*CP*	0.25 (0.11)	0.17 (0.09)	0.28 (0.12)	0.20 (0.10)	0.33 (0.13)	0.25 (0.13)
	*TD*	0.08 (0.03)	0.07 (0.03)	0.10 (0.05)	0.08 (0.03)	0.14 (0.06)	0.10 (0.05)
CCI_F&W_	*CP*	96.58 (14.68)	92.21 (32.13)	98.29 (14.06)	92.20 (30.08)	98.10 (12.33)	91.53 (29.42)
	*TD*	79.59 (9.83)	68.77 (13.02)	82.58 (9.36)	67.46 (10.97)	82.40 (10.62)	64.93 (13.76)

Slow, 75% self-selected walking speed; Normal, 100% self-selected walking speed; Fast, 125% self-selected walking speed; Dry, conventional; Wet, aquatic; CCI_Unnithan_, co-contraction index using Unnithan method; CCI_Rudolph_, co-contraction index using Rudolph method; CCI_F&W_, co-contraction index using Falconer and Winter method; CP, cerebral palsy; TD, typically developing.

**Table 3:** Outcomes of linear mixed-effects models with increasing complexity for each CCI method and muscle pairings.

Models	RF/ST			TA/MG		
	CCI_Unnithan_	CCI_Rudolph_	CCI_F&W_	CCI_Unnithan_	CCI_Rudolph_	CCI_F&W_
CCI ~ 1 + (1 | Subject)	–	–	–	–	–	–
CCI ~ 1 + Environment + (1 | Subject)	*p* < 0.001	*p* < 0.001	*p* = 0.318	*p* < 0.001	*p* < 0.001	*p* < 0.001
CCI ~ 1 + Environment + Population + (1 | Subject)	*p* = 0.296	*p* = 0.294	*p* = 0.342	*p* < 0.001	*p* < 0.001	*p* < 0.001
CCI ~ 1 + Environment + Population + Speed + (1 | Subject)	*p* < 0.001	*p* < 0.001	*p* = 0.783	*p* < 0.001	*p* < 0.001	*p* = 0.776
CCI ~ 1 + Environment + Population + Speed + Environment:Population + (1 | Subject)	*p* < 0.001	*p* < 0.001	***p* = 0.014** ^**Τ**^	*p* < 0.001	*p* < 0.001	***p* < 0.001** ^**Τ**^
CCI ~ 1 + Environment + Population + Speed + Environment:Population + Environment:Speed + (1 | Subject)	***p* = 0.003** ^**Τ**^	***p* < 0.001** ^**Τ**^	*p* = 0.781	*p* = 0.675	*p* = 0.553	*p* = 0.232
CCI ~ 1 + Environment + Population + Speed + Environment:Population + Environment:Speed + Population:Speed (1 | Subject)	*p* = 0.816	*p* = 0.865	*p* = 0.587	***p* = 0.046** ^**Τ**^	***p* = 0.020** ^**Τ**^	*p* = 0.949
CCI ~ 1 + Environment + Population + Speed + Environment:Population + Environment:Speed + Population:Speed + Environment:Population:Speed (1 | Subject)	*p* = 0.447	*p* = 0.381	*p* = 0.845	*p* = 0.768	*p* = 0.790	*p* = 0.782

Τ indicates the most complex model with statistical significance. Bold values in this table are those with special characters ‘T’. This was done to help visualize those values.

CCI_Unnithan_, co-contraction index using the Unnithan method; CCI_Rudolph_, co-contraction index using the Rudolph method; CCI_F&W_, co-contraction index using the Falconer and Winter method; RF is rectus femoris; ST is semitendinosus; TA is tibialis anterior; and MG is medial gastrocnemius.

### 3.1 RF/ST muscle pairing

#### 3.1.1 Method 1—Unnithan

**Figure 2:**
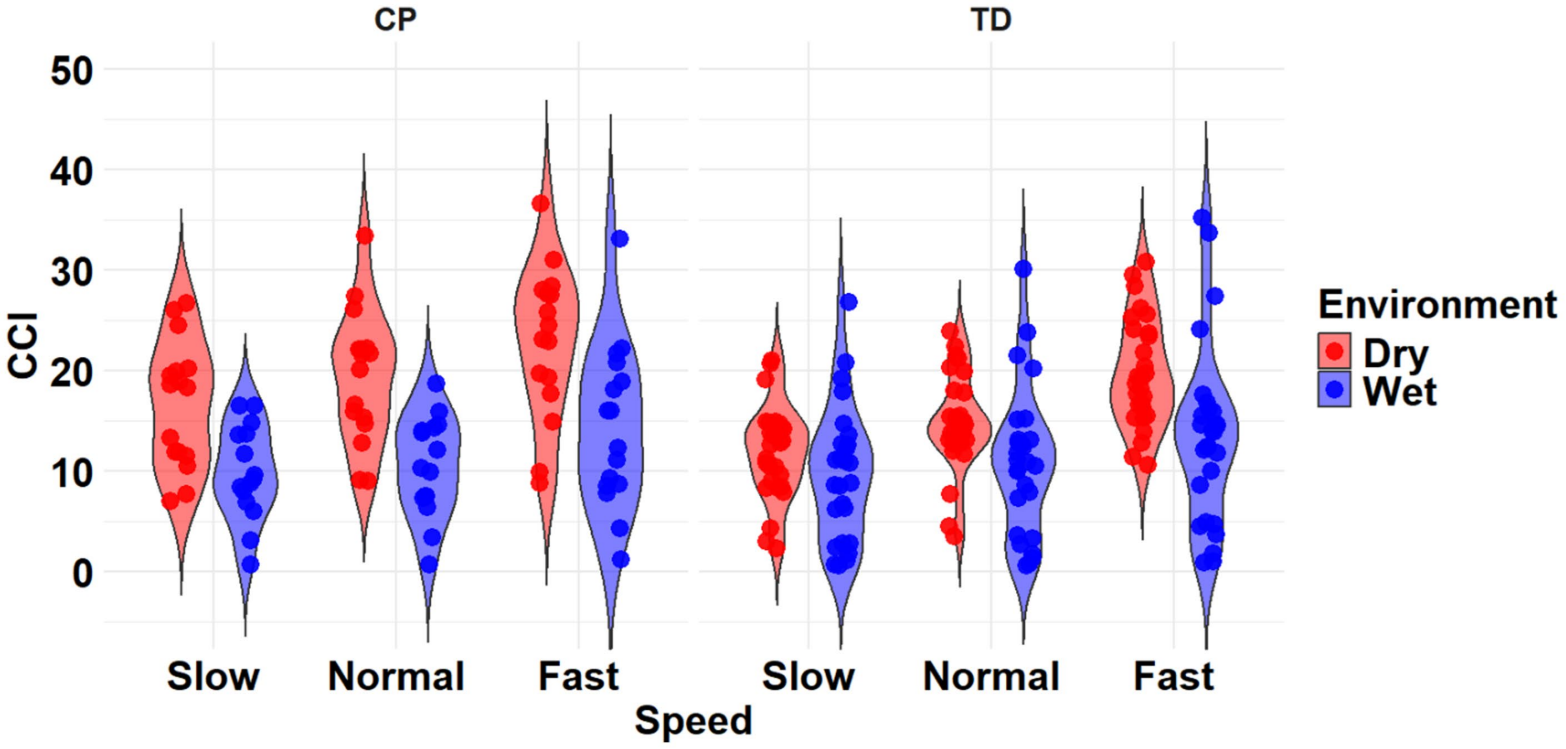
Violin plot of the mean stride-normalized co-contraction index (CCI) using the Unnithan method for the RF/ST muscle pairing of children with CP (left) and TD children (right) during Dry (red) and Wet (blue) treadmill walking trials. Speeds are defined in text. Each data point represents one subject.

The most complex statistically significant model had fixed effects for Environment, Population, and Speed, two-way interactions of Environment x Population and Environment x Speed, along with random intercepts for each subject (AIC = 1502.1, BIC = 1537.6, log-likelihood = −741.03, *p* = 0.003) for the RF/ST muscle pairing (Table 3). Further ANOVA testing of the model revealed significant main effects of Environment (*p* < 0.001) and Speed (*p* < 0.001), as well as significant interaction effects of Environment x Population (*p* < 0.001) and Environment x Speed (*p* = 0.003; Table 4). Pairwise comparisons revealed there were significant differences within children with CP (*p* < 0.001) and TD children (*p* = 0.012) when comparing co-contraction during Dry and Wet treadmill walking (Figure 2). On average, co-contraction was reduced by 41.3% for children with CP and 27.0% for TD children during Wet treadmill walking compared to Dry treadmill walking (Table 1). All Environment x Speed pairwise comparisons revealed there were significant differences in co-contraction within each treadmill environment (*p* < 0.001), except for the Slow-Normal comparison during Wet treadmill walking (*p* = 0.423). When walking on a Dry treadmill, changing speeds from Slow to Normal elicited a 20.3% increase, Normal to Fast elicited a 24.4% increase, and Slow to Fast elicited a 49.7% increase in muscle co-contraction. Furthermore, walking on a Wet treadmill changing speeds from Normal to Fast elicited a 30.9% increase, and Slow to Fast elicited a 42.2% increase in muscle co-contraction.

**Table 4:** ANOVA outcomes of the most complex linear mixed-effects model for each CCI method for the RF/ST muscle pairing.

Variable	Effect	η^2^	*p*
CCI_Unnithan_	Environment	0.52	< 0.001
	Population	0.02	0.349
	Speed	0.38	< 0.001
	Environment x Population	0.10	< 0.001
	Environment x Speed	0.05	0.003
	Population x Speed	–	–
	Environment x Population x Speed	–	–
CCI_Rudolph_	Environment	0.58	< 0.001
	Population	0.02	0.345
	Speed	0.36	< 0.001
	Environment x Population	0.09	< 0.001
	Environment x Speed	0.07	< 0.001
	Population x Speed	–	–
	Environment x Population x Speed	–	–
CCI_F&W_	Environment	0.0005	0.750
	Population	0.02	0.352
	Speed	0.002	0.781
	Environment x Population	0.03	0.015
	Environment x Speed	–	–
	Population x Speed	–	–
	Environment x Population x Speed	–	–

See Table 3 for specifics for each model. η^2^, partial eta-squared effect size; *p*, *p* value; CCI_Unnithan_, co-contraction index using Unnithan method; CCI_Rudolph_, co-contraction index using Rudolph method; CCI_F&W_, co-contraction index using Falconer and Winter method.

#### 3.1.2 Method 2—Rudolph

**Figure 3:**
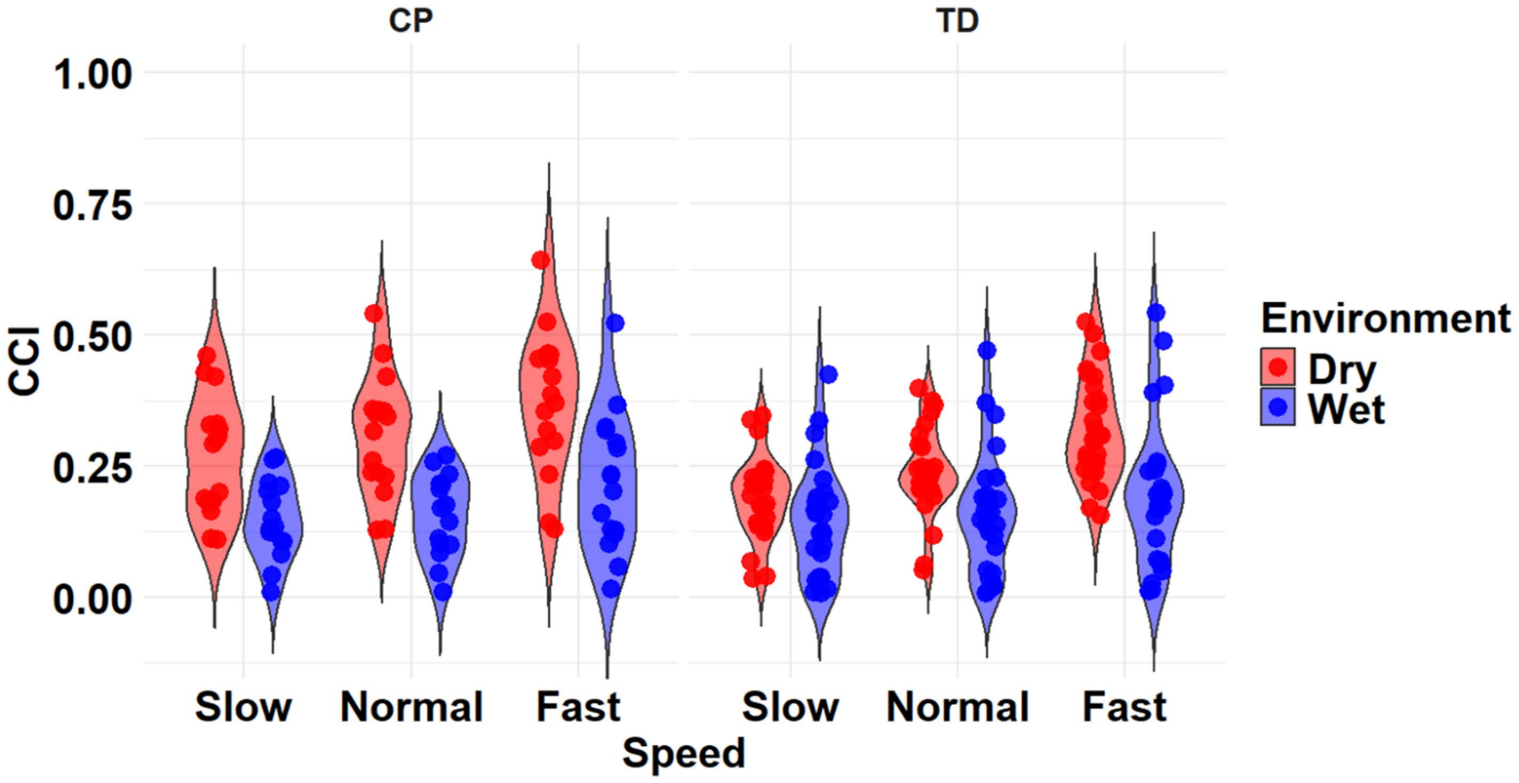
Violin plot of the mean stride-normalized co-contraction index (CCI) using the Rudolph method for the RF/ST muscle pairing of children with CP (left) and TD children (right) during Dry (red) and Wet (blue) treadmill walking trials. Speeds are defined in text. Each data point represents one subject.

The most complex statistically significant model had fixed effects for Environment, Population, and Speed, two-way interactions of Environment x Population and Environment x Speed, along with random intercepts for each subject (AIC = −634.59, BIC = −599.02, log-likelihood = 327.29, *p* < 0.001) for the RF/ST muscle pairing (Table 3). Further ANOVA testing of the model revealed significant main effects of Environment (*p* < 0.001) and Speed (*p* < 0.001), as well as significant interaction effects of Environment x Population (*p* < 0.001) and Environment x Speed (*p* < 0.001; Table 4). Pairwise comparisons revealed there were significant differences within children with CP (*p* < 0.001) and TD children (*p* < 0.001) when comparing co-contraction during Dry and Wet treadmill walking (Figure 3). On average, co-contraction was reduced by 44.2% for children with CP and 32% for TD children during Wet treadmill walking compared to Dry treadmill walking (Table 1). All Environment x Speed pairwise comparisons revealed there were significant differences in co-contraction within each treadmill environment (*p* < 0.001), except for the Slow-Normal comparison during Wet treadmill walking (*p* = 0.610). Walking on a Dry treadmill changing speeds from Slow to Normal elicited a 19.6% increase, Normal to Fast elicited a 25.5% increase, and Slow to Fast elicited a 50.0% increase in muscle co-contraction. Furthermore, walking on a Wet treadmill changing speeds from Normal to Fast elicited a 31.3% increase, and Slow to Fast elicited a 40.0% increase in muscle co-contraction.

#### 3.1.3 Method 3—Falconer and Winter

**Figure 4:**
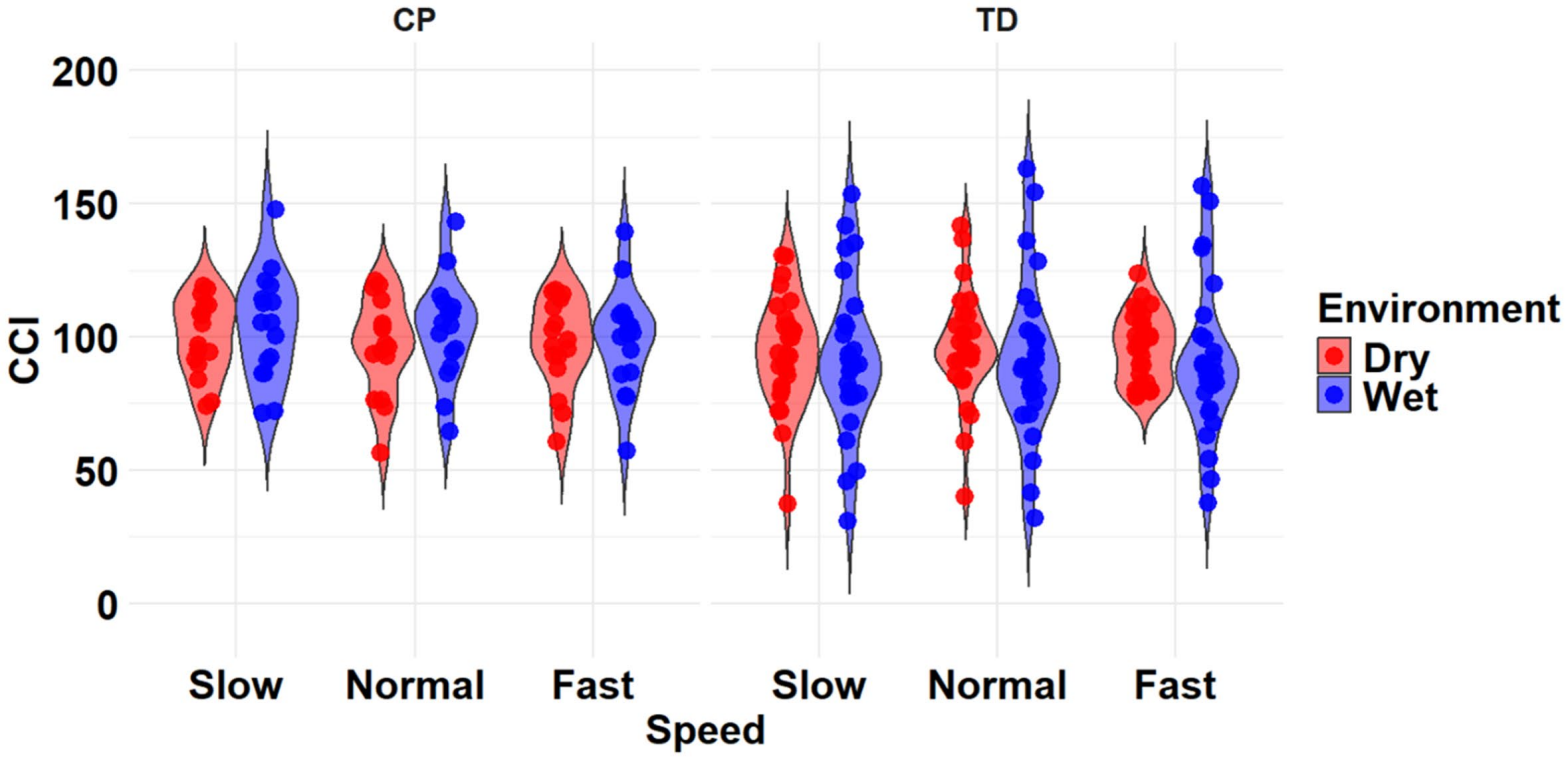
Violin plot of the mean stride-normalized co-contraction index (CCI) using the method from Falconer and Winter for the RF/ST muscle pairing of children with CP (left) and TD children (right) during Dry (red) and Wet (blue) treadmill walking trials. Speeds are defined in text. Each data point represents one subject.

The most complex statistically significant model had fixed effects for Environment, Population, and Speed, a two-way interaction (Environment x Population), along with random intercepts for each subject (AIC = 2212.5, BIC = 2241.0, log-likelihood = −1098.2, *p* = 0.014) for the RF/ST muscle pairing (Table 3). Further ANOVA testing of the model revealed non-significant main effects of Environment (*p* = 0.750), Population (*p* = 0.352), and Speed (*p* = 0.781), and a significant interaction effect of Environment x Population (*p* = 0.015; Table 4). Pairwise comparisons revealed a non-significant difference within children with CP (*p* = 0.181) and a significant difference within TD children (*p* = 0.023) when comparing co-contraction during Dry and Wet treadmill walking (Figure 4). On average, TD children had 4.1% lower co-contraction during Wet treadmill walking compared to Dry treadmill walking (Table 1).

### 3.2 TA/MG muscle pairing

#### 3.2.1 Method 1—Unnithan

**Figure 5:**
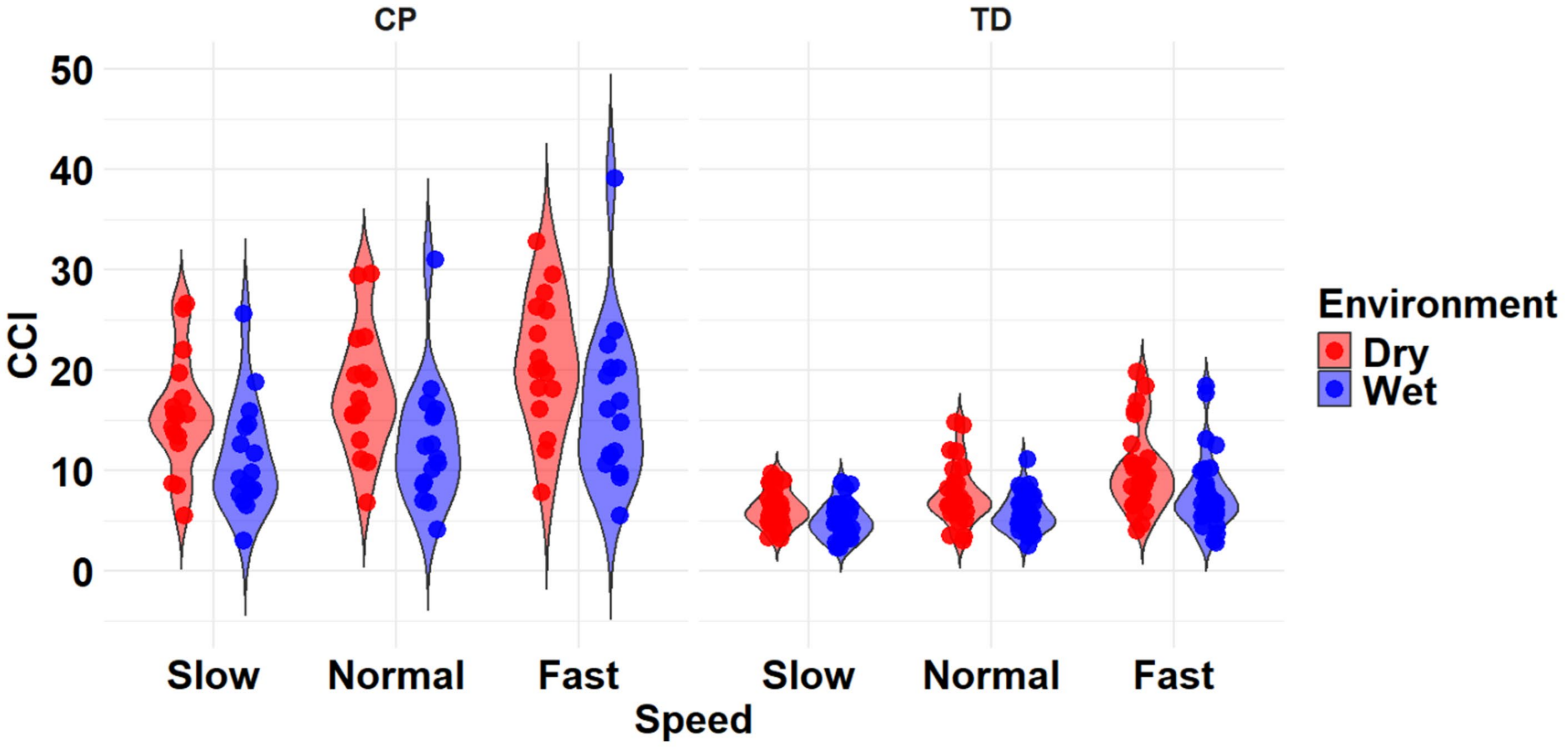
Violin plot of the mean stride-normalized co-contraction index (CCI) using the Unnithan method for the TA/MG muscle pairing of children with CP (left) and TD children (right) during Dry (red) and Wet (blue) treadmill walking trials. Speeds are defined in text. Each data point represents one subject.

The most complex statistically significant model had fixed effects for Environment, Population, and Speed, their two-way interactions, along with random intercepts for each subject (AIC = 1388.8, BIC = 1432.1, log-likelihood = −682.42, *p* = 0.046) for the TA/MG muscle pairing (Table 3). Further ANOVA testing of the model revealed significant main effects of Environment (*p* < 0.001), Population (*p* < 0.001), and Speed (*p* < 0.001), as well as a significant interaction effect of Environment x Population (*p* < 0.001; Table 5). Pairwise comparisons revealed there were significant differences within children with CP (*p* < 0.001) and TD children (*p* < 0.001) when comparing co-contraction during Dry and Wet treadmill walking (Figure 5). On average, co-contraction was reduced by 25.3% for children with CP and 21.9% for TD children during Wet treadmill walking compared to Dry treadmill walking (Table 2). All pairwise comparisons of Speed, averaged over Environment and Population, were significantly different (*p* < 0.001). Walking on a treadmill changing speeds from Slow to Normal elicited a 15.0% increase, Normal to Fast elicited a 24.9% increase, and Slow to Fast elicited a 43.6% increase in muscle co-contraction.

**Table 5:** ANOVA outcomes of the most complex linear mixed-effects model for each CCI method for the TA/MG muscle pairing.

Variable	Effect	η^2^	*p*
CCI_Unnithan_	Environment	0.31	< 0.001
	Population	0.56	< 0.001
	Speed	0.37	< 0.001
	Environment x Population	0.08	< 0.001
	Environment x Speed	0.0036	0.680
	Population x Speed	0.03	0.051
	Environment x Population x Speed	–	–
CCI_Rudolph_	Environment	0.33	< 0.001
	Population	0.57	< 0.001
	Speed	0.33	< 0.001
	Environment x Population	0.13	< 0.001
	Environment x Speed	0.0055	0.552
	Population x Speed	0.03	0.023
	Environment x Population x Speed	–	–
CCI_F&W_	Environment	0.21	< 0.001
	Population	0.37	< 0.001
	Speed	0.0023	0.776
	Environment x Population	0.05	< 0.001
	Environment x Speed	–	–
	Population x Speed	–	–
	Environment x Population x Speed	–	–

See Table 3 for specifics of each model. η^2^, partial eta-squared effect size; *p*, *p* value; CCI_Unnithan_, co-contraction index using Unnithan method; CCI_Rudolph_, co-contraction index using Rudolph method; CCI_F&W_, co-contraction index using Falconer and Winter method.

#### 3.2.2 Method 2—Rudolph

**Figure 6:**
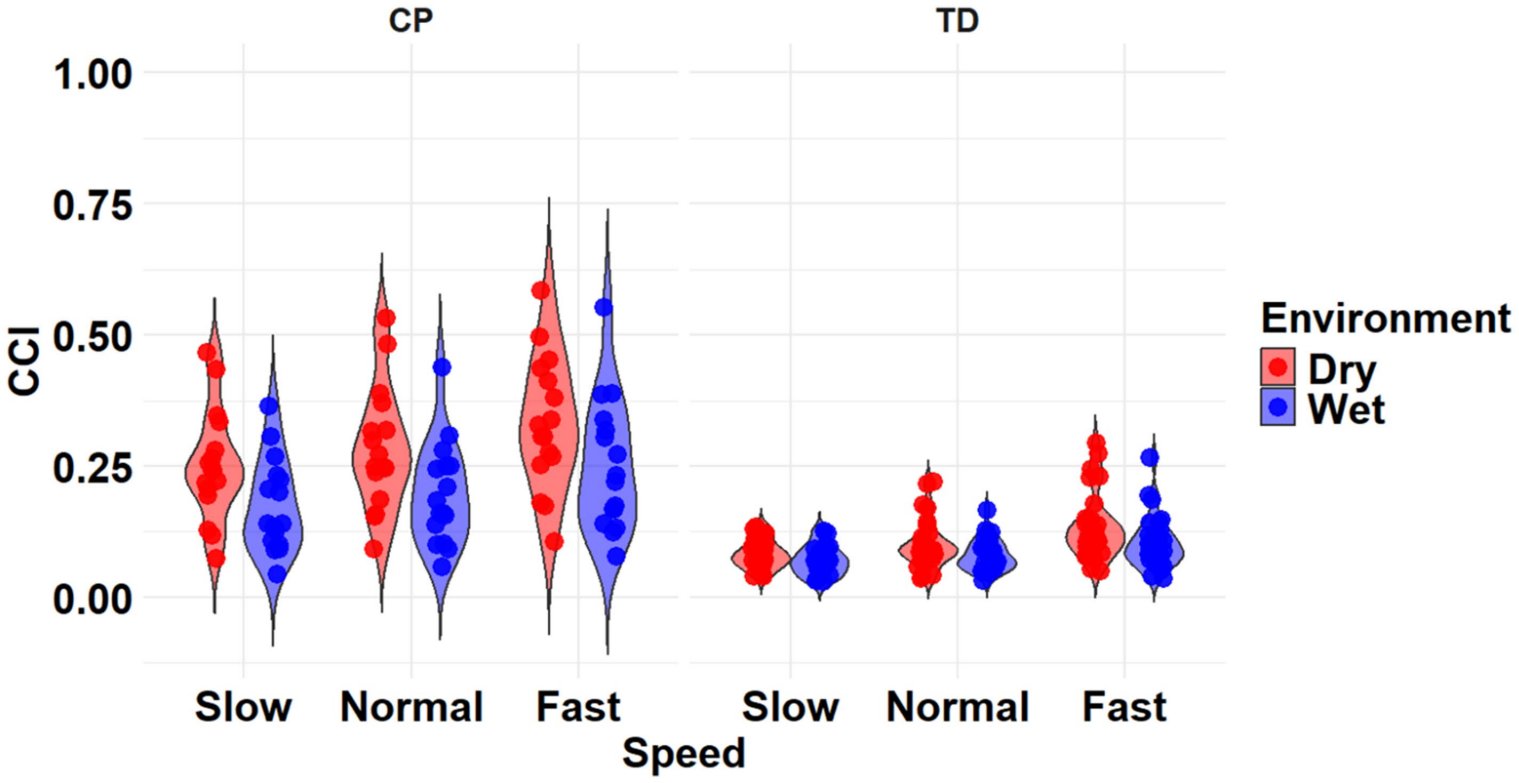
Violin plot of the mean stride-normalized co-contraction index (CCI) using the method from Rudolph for the TA/MG muscle pairing of children with CP (left) and TD children (right) during Dry (red) and Wet (blue) treadmill walking trials. Speeds are defined in text. Each data point represents one subject.

The most complex statistically significant model had fixed effects for Environment, Population, and Speed, their two-way interactions, along with random intercepts for each subject (AIC = −848.60, BIC = −805.42, log-likelihood = 436.30, *p* = 0.020) for the TA/MG muscle pairing (Table 3). Further ANOVA testing of the model revealed significant main effects of Environment (*p* < 0.001), Population (*p* < 0.001), and Speed (*p* < 0.001), as well as significant interaction effects of Environment x Population (*p* < 0.001) and Population x Speed (*p* = 0.023; Table 5). Pairwise comparisons revealed there were significant differences within children with CP (*p* < 0.001) and TD children (*p* = 0.002) when comparing co-contraction during Dry and Wet treadmill walking (Figure 6). On average, co-contraction was reduced by 27.9% for children with CP and 21.9% for TD children during Wet treadmill walking compared to Dry treadmill walking (Table 2). All Environment x Speed pairwise comparisons revealed there were significant differences in co-contraction within each population (*p* < 0.013), except for the Slow-Normal comparison for TD children (*p* = 0.102). During treadmill walking, children with CP elicited a 15.2% increase, 23.7% increase, and a 42.4% increase changing speeds from Slow to Normal, Normal to Fast, and Slow to Fast, respectively. Furthermore, during treadmill walking TD children elicited a 25.0% increase and 45.8% increase when changing speeds from Normal to Fast and Slow to Fast, respectively.

#### 3.2.3 Method 3—Falconer and Winter

**Figure 7:**
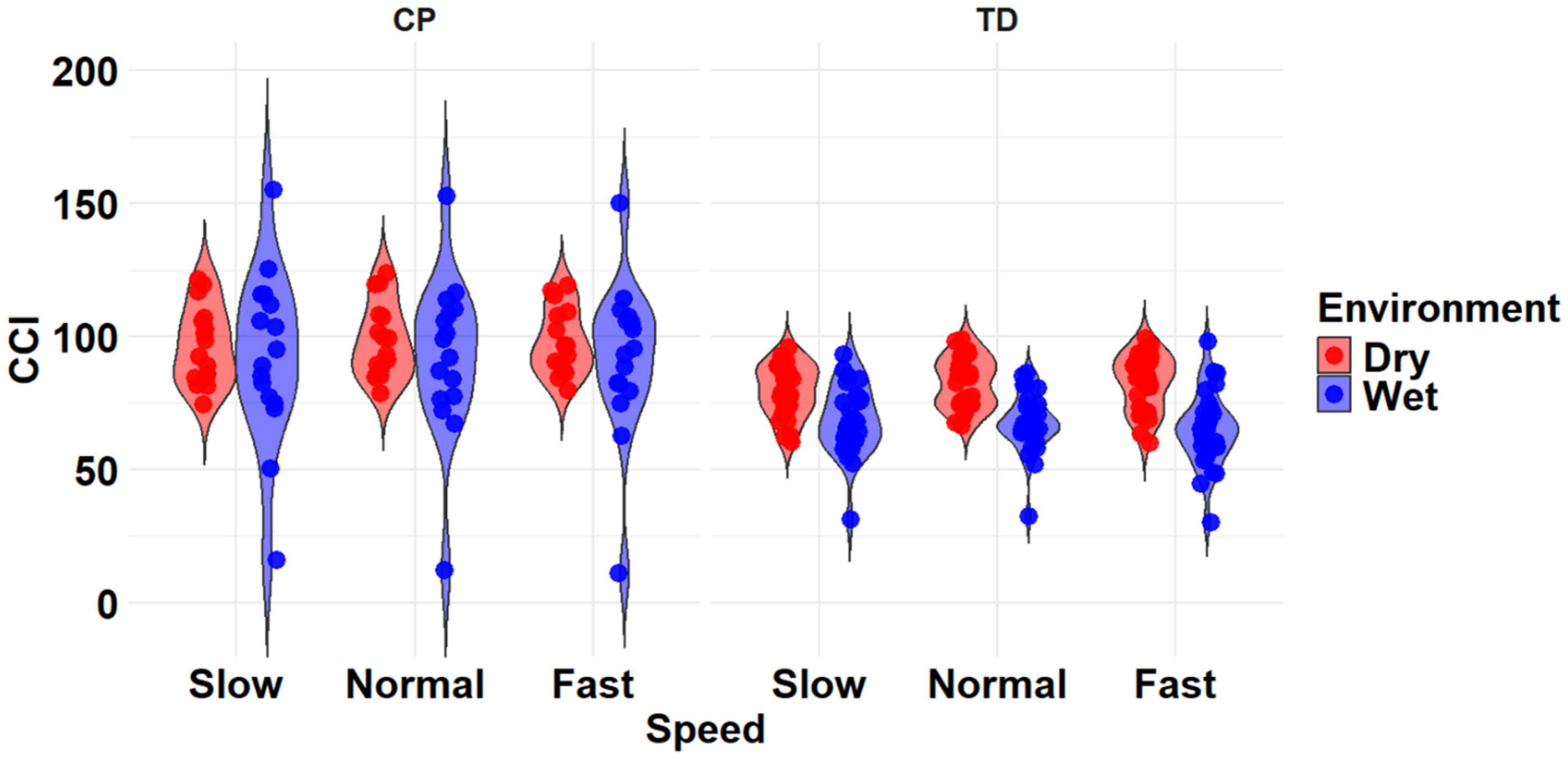
Violin plot of the mean stride-normalized co-contraction index (CCI) using the method from Falconer and Winter for the TA/MG muscle pairing of children with CP (left) and TD children (right) during Dry (red) and Wet (blue) treadmill walking trials. Speeds are defined in text. Each data point represents one subject.

The most complex statistically significant model had fixed effects for Environment, Population, and Speed, a two-way interaction (Environment x Population), along with random intercepts for each subject (AIC = 2146.7, BIC = 2175.5, log-likelihood = −1065.3, *p* < 0.001) for the TA/MG muscle pairing (Table 3). Further ANOVA testing of the model revealed significant main effects of Environment (*p* < 0.001) and Population (*p* < 0.001), a non-significant main effect of Speed (*p* = 0.776), and a significant interaction effect of Environment x Population (*p* = 0.002; Table 5). Pairwise comparisons revealed there were significant differences within children with CP (*p* = 0.009) and TD children (*p* < 0.001) when comparing co-contraction during Dry and Wet treadmill walking (Figure 7). On average, co-contraction was reduced by 5.8% for children with CP and 17.7% for TD children during Wet treadmill walking compared to Dry treadmill walking (Table 2).

## 4 Discussion

The purpose of this study was to investigate muscle co-contraction during Wet and Dry treadmill walking in TD and children with CP using three common CCI methods. Additionally, we explored how the clinical interpretation of co-contraction may change depending on the CCI method used. Overall, our first hypothesis was partially supported as Wet treadmill walking reduced muscle co-contraction in TD (CCI_Unnithan_, CCI_Rudolph_, and CCI_F&W_) and children with CP (CCI_Unnithan_ and CCI_Rudolph_) for the RF/ST muscle pairing and the TA/MG muscle pairing, according to all CCI methods used. Partially supporting our second hypothesis, Fast treadmill walking produced the greatest muscle co-contraction when using CCI_Unnithan_ and CCI_Rudolph_, while when using CCI_F&W_, speed had no effect on muscle co-contraction, regardless of muscle pairing.

In the present study, several comparisons for muscle co-contraction between treadmill walking at Slow and Normal speeds did not reach statistical significance. Given that successful partial body weight support rehabilitation programs typically involve 20 to 30 min of walking (37, 38), slower speeds may enhance the feasibility of completing sessions where trained observers can provide feedback and encouragement. The reduced effect of speed on co-contraction may indicate that Wet treadmill training could offer a more favorable environment in the early stages of rehabilitation for individuals with impairments. Our results suggest that children could train in water at various speeds (slow to moderate) without significant increases in thigh muscle co-contraction, likely reducing the chance of local muscular fatigue and providing an environment where children can better focus on gait mechanics and motor control (10, 39). After motor control during gait is established in the water, Dry treadmill training could be used to further develop motor skills, progressively challenging the neuromuscular system and improving independence in daily activities. While the lack of significant differences in co-contraction between slower speeds in water might initially appear inconsequential, it underscores the potential of aquatic training programs for rehabilitation.

To our knowledge, this is the first study to investigate lower extremity muscle co-contraction during Dry and Wet treadmill walking in TD and children with CP. Our findings demonstrate that Wet treadmill walking led to a reduction in co-contraction of thigh musculature, ranging between 4.1 to 32% in TD children and approximately 40% in children with CP across the gait cycle. Similarly, co-contraction of the shank musculature was reduced by approximately 20% in TD children and between 5.8 to 27.9% in children with CP. These findings partially align with previous research investigating overground and pool-based walking in TD and children with CP that reported lower co-contraction of thigh muscles and an increase in shank muscles during pool-based walking in children with CP (11). The reduction in thigh musculature co-contraction observed in the present study is speculated to result from the buoyancy of water. Body weight support resulting from buoyancy is speculated to reduce the need for stabilization around the hip and knee joints, which may allow for a more relaxed gait pattern in water. In contrast, the present study observed a reduction in shank musculature co-contraction, which may have resulted from a combination of factors, including buoyancy, hydrodynamic drag, differences in immersion level, walking speed, length of walking trials, the walking surface (pool floor vs. treadmill), the use of handrails, as well as the specific CCI method employed. Each of these variables could influence gait patterns and muscle activation (7, 8, 12, 40–53); however, the CCI method significantly highlights the complexity of comparing results across studies (13, 14, 54–56).

Each CCI calculation method is inherently different, as some use the magnitude of muscle activation to distinguish the location in the calculation while others only consider if the muscle is the antagonist or the agonist during a specific movement or task. CCI_Unnithan_, defined as the integral of the minimum values of the agonist and antagonist EMG signals over time, represents the area of overlap between the two signals, capturing both the intensity and duration of muscle co-contraction (20). CCI_Rudolph_ measures the relative timing of a muscle pair in addition to the magnitude of co-contraction, where low co-contraction values represent greater selective muscle activation and high co-contraction values represent more generalized muscle activation (21). CCI_F&W_ measures antagonist muscle activation relative to total muscle activation (16). Two co-contraction methods used in the present study, CCI_Unnithan_ and CCI_Rudolph_ consider how the activation magnitudes change over the gait cycle (a combination of magnitude and time). This may be visually supported by Figure 1, as CCI_Unnithan_ and CCI_Rudolph_ have similar waveform shapes within environments for both populations while CCI_F&W_ has a different shape entirely. Therefore, CCI_F&W_ may oversimplify co-contraction by focusing on one muscle’s magnitude and not accounting for the timing of the muscle activation.

Muscle co-contraction using a modified CCI_Unnithan_ (54) and CCI_F&W_ has been reported in young, healthy adults during overground walking (54). It was found that CCI_F&W_ produced larger co-contraction values than the modified CCI_Unnithan_ for muscles around the knee and ankle during gait. While the Integration method was not modified in the current study, our results agree that the interpretation changes depending on the methodology used. However, Gagnat and colleagues found that muscle co-contraction calculated using CCI_F&W_ and the Ikeda method (the ratio between the magnitude of the antagonist muscle and maximum EMG) produced the same significant deviations between children with CP and TD children during overground walking, although the Ikeda values were larger (55). Due to the exploratory nature of determining the influence of CCI method in Wet treadmill walking, the Ikeda method was not chosen in the present study due to its rarity of use in current literature. Larger co-contraction values using CCI_Unnithan_ indicate more simultaneous contraction between the agonist and antagonist muscles, while larger co-contraction values using CCI_F&W_ indicate a greater proportion of total muscle activation is attributed to the antagonist muscle. Additionally, Li and colleagues investigated the feasibility of CCI_Rudolph_ and CCI_F&W_ to approximate lower limb joint stiffness during gait (14). They found that CCI_Rudolph_ was more correlated with joint stiffness than CCI_F&W_ and speculated that this resulted from CCI_F&W_ only measuring antagonist muscle activation relative to total muscle activation. These studies highlight that caution is needed when comparing CCI values, as the method used and normalization techniques can significantly influence the interpretation (29, 54–56).

This study is not without its limitations. Due to the small sample size of children with CP, we could not dissociate co-contraction between different GMFCS levels. Such future efforts will provide insight into how aquatic treadmill walking affects children with varying impairments of motor abilities. Additionally, the wide range of calculation and EMG normalization methods for co-contraction makes comparisons to the existing literature difficult. Therefore, our discussion was primarily based on comparing the interpretations of co-contraction methods during gait rather than the numerical outcomes. However, while there is no consensus on the best approach (to normalize, and if so how), a dynamic approach is common in the literature. For instance, dynamically normalizing EMG was used in the articles that first proposed CCI_Unnithan_ (20) and CCI_Rudolph_ (21), as well as others that have calculated CCI (22, 28–30). Lastly, we could not accurately separate the gait cycle into distinct sub-phases (e.g., loading response, midstance, terminal stance, etc.) using our IMU data; therefore, we only assessed muscle co-contraction over the entire gait cycle. Splitting the gait cycle into sub-phases would provide additional information on CCI timing and could change interpretations or rehabilitative approaches (e.g., neuromuscular electrical stimulation) that rely on gait timing as an input parameter.

## 5 Conclusion

Aquatic treadmill walking reduced muscle CCI in TD and CP populations, highlighting the potential importance of walking environment considerations in rehabilitation. Tailored interventions using an aquatic treadmill environment may help optimize gait outcomes in clinical populations that have challenges with lower-extremity muscle weakness, increased muscle co-contraction, and spasticity. However, caution is advised when comparing muscle CCI values across studies, as different methods can produce different results, impacting clinical implications. Our investigation may suggest that CCI_Unnithan_ has the advantage of accounting for the magnitude and timing of the muscle, regardless of when a particular muscle ‘should’ have activated in typical gait. Therefore, it may be more appropriate for studies determining muscle co-contraction during any movement or task to report values from multiple methods.

## Data Availability

The raw data supporting the conclusions of this article will be made available by the authors, without undue reservation.
